# ERK5 Phosphorylates K_v_4.2 and Inhibits Inactivation of the A-Type Current in PC12 Cells

**DOI:** 10.3390/ijms19072008

**Published:** 2018-07-10

**Authors:** Yurina Kashino, Yutaro Obara, Yosuke Okamoto, Takeo Saneyoshi, Yasunori Hayashi, Kuniaki Ishii

**Affiliations:** 1 Department of Pharmacology, Yamagata University School of Medicine, Yamagata 990-9585, Japan; yurikamo_chronicle@yahoo.co.jp (Y.K.); okamoto@med.akita-u.ac.jp (Y.O.); kuishii@med.id.yamagata-u.ac.jp (K.I.); 2 Department of Pharmacology, Kyoto University Graduate School of Medicine, Kyoto 606-8501, Japan; saneyoshi.takeo.3v@kyoto-u.ac.jp (T.S.); yhayashi-tky@umin.ac.jp (Y.H.)

**Keywords:** extracellular signal-regulated kinase 5 (ERK5), K_v_4.2, PC12 cells

## Abstract

Extracellular signal-regulated kinase 5 (ERK5) regulates diverse physiological responses such as proliferation, differentiation, and gene expression. Previously, we demonstrated that ERK5 is essential for neurite outgrowth and catecholamine biosynthesis in PC12 cells and sympathetic neurons. However, it remains unclear how ERK5 regulates the activity of ion channels, which are important for membrane excitability. Thus, we examined the effect of ERK5 on the ion channel activity in the PC12 cells that overexpress both ERK5 and the constitutively active MEK5 mutant. The gene and protein expression levels of voltage-dependent Ca^2+^ and K^+^ channels were determined by RT-qPCR or Western blotting. The A-type K^+^ current was recorded using the whole-cell patch clamp method. In these ERK5-activated cells, the gene expression levels of voltage-dependent L- and P/Q-type Ca^2+^ channels did not alter, but the N-type Ca^2+^ channel was slightly reduced. In contrast, those of K_v_4.2 and K_v_4.3, which are components of the A-type current, were significantly enhanced. Unexpectedly, the protein levels of K_v_4.2 were not elevated by ERK5 activation, but the phosphorylation levels were increased by ERK5 activation. By electrophysiological analysis, the inactivation time constant of the A-type current was prolonged by ERK5 activation, without changes in the peak current. Taken together, ERK5 inhibits an inactivation of the A-type current by phosphorylation of K_v_4.2, which may contribute to the neuronal differentiation process.

## 1. Introduction

Conventional mitogen-activated protein kinases (MAPKs) involve extracellular signal-regulated kinases (ERKs) 1, 2, and 5, c-Jun N-terminal kinase and p38 MAPKs, and atypical MAPKs include ERK3, 4, and 7 and nemo-like kinase [1]. In response to growth factors or neurotrophic factors, ERKs are strongly activated and regulate diverse physiological responses, such as proliferation, differentiation, and gene expression. The signal transduction leading to ERK1/2 activation and the involvement of ERK1/2 in cellular responses are the best studied among the MAPK family members.

ERK5 shares homology in the amino acid sequence in the kinase-domain with ERK1/2, and possesses a unique long C-terminal domain [2,3]. In the past 10 years, specific inhibitors of ERK5 signaling, such as BIX02189 [4,5] and XMD8-92 [6], have been developed. Using these pharmacological inhibitors, the role of ERK5 in tumor genesis and metastatic progression has been especially well understood [7,8]. We have shown that the levels of ERK5 and tyrosine hydroxylase, a rate-limiting enzyme for catecholamine biosynthesis, are co-related in normal human adrenal medulla, but this correlation is disrupted in pheochromocytomas [9]. However, signaling pathways for ERK5 activation and physiological roles of ERK5 in neuronal development are relatively unclear. For example, involvements of small G-proteins in ERK5 activation are vague [10], whereas it has been established that ERK1/2 is activated through Ras and Rap1 [11,12]. Some limited studies suggest that ERK5 is necessary and sufficient for neuronal differentiation of progenitor cells [13], and is essential for adult hippocampal neurogenesis [14,15]. ERK5 promotes neuronal survival in sympathetic or sensory neurons [16,17]. We have shown that ERK5 is essential for neurite/axon outgrowth and catecholamine biosynthesis in PC12 cells and sympathetic neurons [5,9]. Thus, ERK5 plays important roles in neuronal survival, as well as morphological and functional differentiation. However, although it is well known that ERK1/2 regulates membrane excitability (i.e., neuronal activity) [18,19,20,21], ERK5 regulation of membrane excitability has been poorly understood. Therefore, in the present study, we attempted to clarify the effect of ERK5 signaling on ion channel activity, which is important for regulating membrane excitability.

## 2. Results

**Figure 1:**
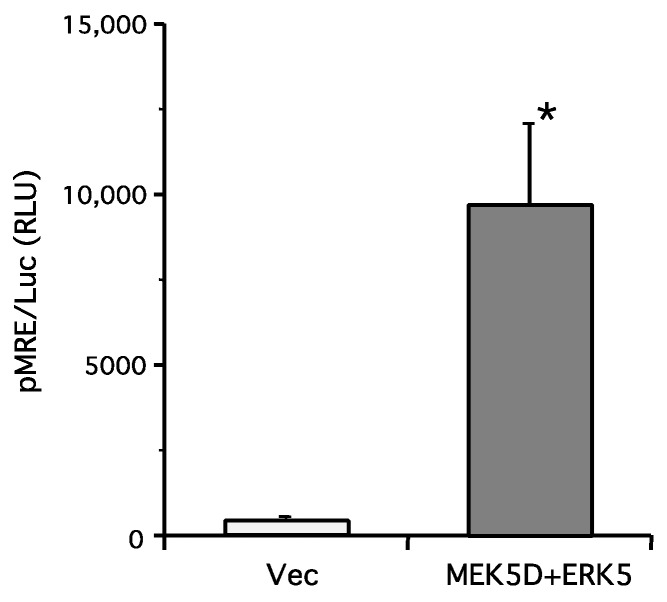
Overexpression of constitutively active mitogen-activated protein and extracellular signal-regulated (MAPK/ERK) kinase (MEK) 5 mutant and ERK5 causes activation of ERK5 signaling. Human embryonic kidney 293 cells (HEK293 cells) were transfected with tandem myocyte-enhancer factor (MEF) 2 response element (MRE)-luciferase reporter gene and empty vector (Vec) or MEK5D and ERK5. Two days after transfection, the luciferase activity resulting from MEF2 activation was measured. ERK5 significantly increased MEF2C activity (one experiment in triplicate (*n* = 3), * *p* <0.05, unpaired Student’s *t*-test).

To examine the effect of ERK5 signaling, we attempted to activate ERK5 selectively by the overexpression of ERK5 wildtype and a constitutively active mutant of MAPK/ERK kinase (MEK) 5 (MEK5S311D/T315D, or MEK5D for short). To confirm that ERK signaling is activated by transfection with these DNA constructs, we measured the myocyte-enhancer factor (MEF) 2 transcriptional activity by reporter gene assay as an index of ERK5 activation. It has been well-established that ERK5 phosphorylates MEF2C directly and the transcriptional activity increases [22]. In human embryonic kidney 293 cells (HEK293 cells), overexpression of MEK5D and ERK5 resulted in a dramatic enhancement of MEF2C activity (Figure 1).

**Figure 2:**
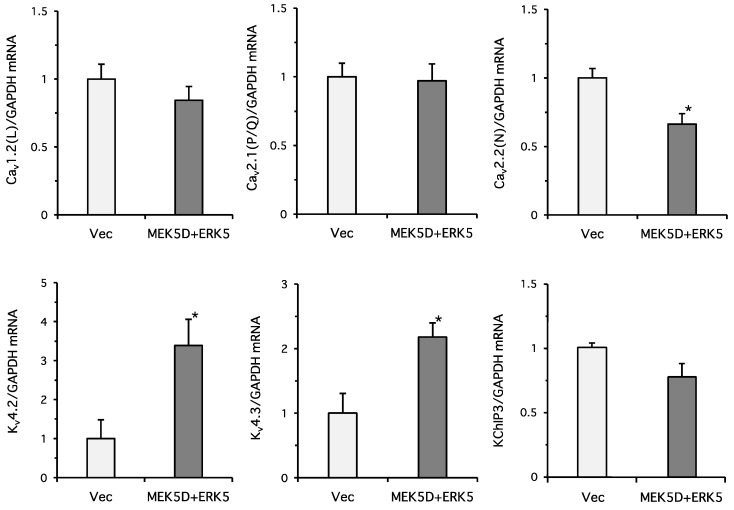
ERK5 promotes gene expression of K_v_4.2 and K_v_4.3 in PC12 cells. PC12 cells were transfected with empty vector (Vec) or MEK5 and ERK5. Two days after transfection, the total RNA was isolated from the cell lysates and RT-qPCR was performed using specific primers for Ca_v_1.2, Ca_v_2.1, Ca_v_2.2, K_v_4.2, K_v_4.3, and KChIP3. ERK5 significantly promoted the gene expression of K_v_4.2 and K_v_4.3, and attenuated Ca_v_2.2 expression (two independent experiments in triplicate (*n* = 6), * *p* <0.05, unpaired Student’s *t*-test).

We previously demonstrated that overexpression of MEK5D and ERK5 strengthens ERK5 signaling, accompanied by the phosphorylation of the Thr-Glu-Tyr (TEY) activation motif and auto-phosphorylation sites on ERK5, but the ERK1/2 TEY phosphorylation site is not affected [23]. Next, PC12 cells were co-transfected with MEK5D and ERK5, and the messenger RNA (mRNA) expression levels of the major voltage-dependent Ca^2+^ and K^+^ channels were measured by RT-qPCR (Figure 2). There were no significant changes in the expression levels of Ca_v_1.2 (L-type) and Ca_v_2.1 (P/Q-type), but a significant reduction of Ca_v_2.2 (N-type) was observed. In contrast, the K_v_4.2 and K_v_4.3 expression levels, which are responsible for the transient outward I_to_ current (A type-current), were significantly promoted by ERK5 signaling. K^+^ channel-interacting proteins (KChIPs) are β-subunit for K_v_4.2, and the A-type current is influenced by the expression of KChiPs [24]. We previously performed RNA-sequencing to examine the gene expression levels comprehensively in PC12 cells [25]. The RPKM values for KChIPs 1, 2, 3, and 4 were 0.021284, 0, 1.21248, and 0.067198, respectively (*n* = 3). Because KChIP3 is a major β-subunit for K_v_4.2 in PC12 cells, we examined the KChIP3 expression levels. But, there was no significant change in the expression levels. It has been shown that overexpression of the RasG12V (RasV12) oncogenic mutant can strongly activate ERK1/2 signaling without affecting the phosphorylation status of the ERK5 TEY motif [5,23]. Constitutive ERK1/2 activation by the overexpression of RasV12 did not elevate the significant expression of K_v_4.2 and K_v_4.3 in our condition (0.791-fold, *n* = 6, *p* = 0.603 for K_v_4.2 and 0.522-fold, *n* = 3, *p* = 0.454 for K_v_4.3). Because K_v_4.2 mediates the majority of the A-type current and is a critical molecule for the modulation of neuronal excitability in many types of neurons, including the cornu ammonis (CA) 1 pyramidal neurons of the hippocampus and the dorsal horn neurons [20,24], we focused on ERK5 regulation of K_v_4.2 for further study. 

**Figure 3:**
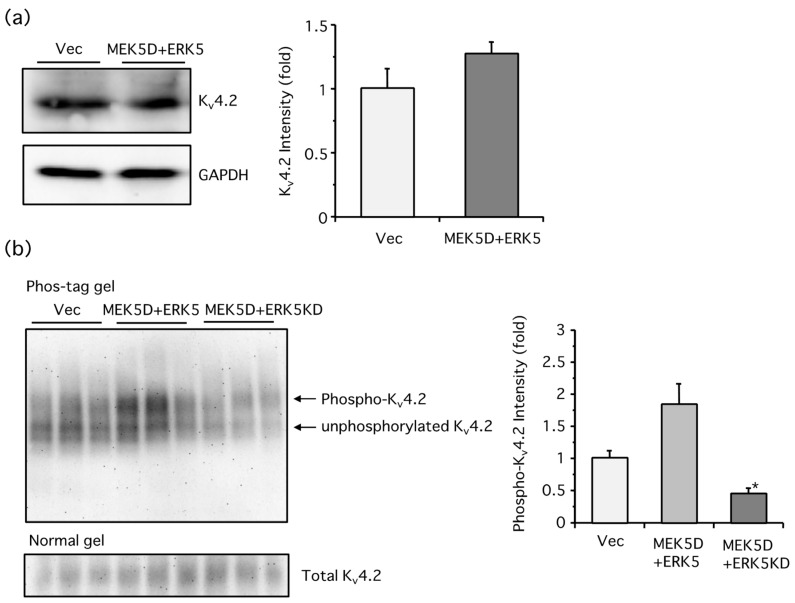
ERK5 did not alter the protein expression of K_v_4.2, but did promote the phosphorylation of K_v_4.2 in PC12 cells. (**a**) PC12 cells were transfected with empty vector (Vec) or MEK5D and ERK5. Two days after transfection, Western blotting using K_v_4.2 and glyceraldehyde-3-phosphate dehydrogenase (GAPDH) antibodies was performed. The density of the K_v_4.2 bands was expressed as a fold of control cells (Vec) (two independent experiments in triplicate (*n* = 6), unpaired Student’s *t*-test); (**b**) PC12 cells were transfected with empty vector (Vec), MEK5D and ERK5, or MEK5D and ERK5KD. Two days after transfection, Western blotting using Phos-tag-contained polyacrylamide gels or Phos-tag-free gels was carried out with a K_v_4.2 antibody. The density of the phospho-K_v_4.2 bands was expressed as a fold of the control cells (Vec). Overexpression of MEK5D and ERK5KD significantly attenuated the MEK5D and ERK5-induced K_v_4.2 phosphorylation (three similar independent experiments in triplicate (*n* = 3), * *p* <0.05, Tukey’s method).

We next examined the protein levels of K_v_4.2 after th eERK5 activation in PC12 cells. Surprisingly, although the mRNA levels were increased, the protein levels were not altered significantly (Figure 3a). In addition to expression levels, we investigated the phosphorylation status of K_v_4.2, because it has been reported that ERK1/2 phosphorylates at least three Ser/Thr residues at the C-terminus of K_v_4.2 and both ERK5 and ERK1/2 preferentially phosphorylate Ser/Thr residues that have a similar minimum consensus sequence (i.e., Ser/Thr-Pro) [24,26,27]. We used a Phos-tag reagent, which tightly binds phosphorylated amino acids in the presence of Mn^2+^ or Zn^2+^. In principle, the Phos-tag-mixed sodium dodecyl sulfate-polyacrylamide gel electrophoresis (SDS-PAGE) causes a band-shift of phosphorylated proteins, and they can be clearly distinguished from unphosphorylated proteins. The overexpression of MEK5D and ERK5 caused the band-shift of K_v_4.2, which was significantly diminished by the dominant-negative ERK5 kinase-dead mutant (ERK5K83M, or ERK5KD for short), suggesting that ERK5 signaling promoted phosphorylation levels of K_v_4.2 (Figure 3b).

**Figure 4:**
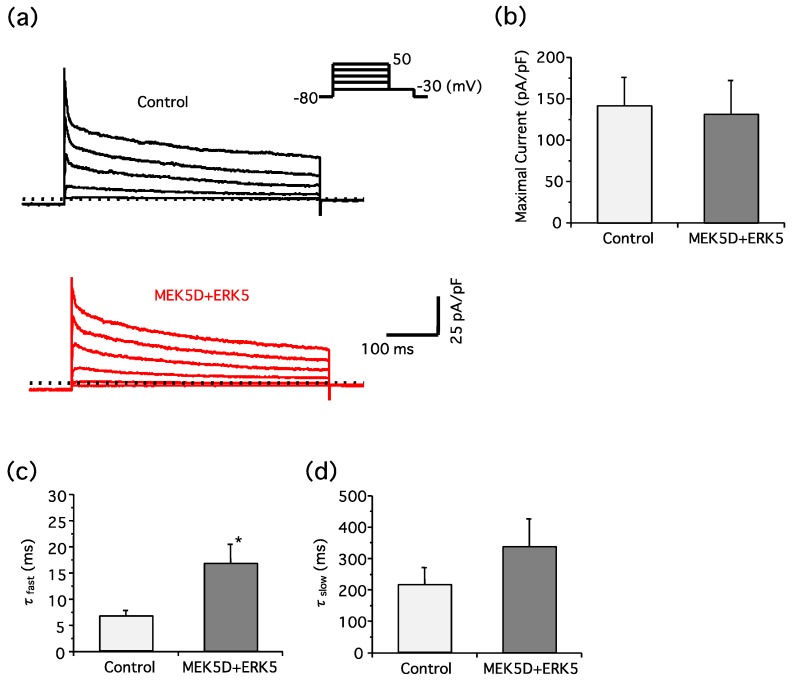
ERK5 inhibits inactivation of the A-type current in PC12 cells. PC12 cells were co-transfected with EGFP, MEK5D, and ERK5. Two days after transfection, the A-type current was recorded. (**a**) Representative traces and step-pulse protocol are shown; (**b**) maximal peak current was measured at +50 mV. The ERK5 did not significantly change amplitude levels (data from three independent experiments (*n* = 4), unpaired Student’s *t*-test); (c,d) the time constant (τ) at fast (**c**) and slow (**d**) phases at +50 mV was calculated. ERK5 significantly changed the time constant (τ) at the fast phase (data from three independent experiments (*n* = 4), * *p* <0.05, unpaired Student’s *t*-test).

We next examined the effect of ERK5 signaling on the A-type current in PC12 cells. The peak current was unchanged in the PC12 cells overexpressing MEK5D and ERK5, but there was a significant slowing of inactivation (Figure 4). The time constant (τ) at the fast and slow phases was 6.663 and 213.0 (ms), respectively, in the control cells, and 16.69 and 334.7 (ms), respectively, in the cells co-transfected with MEK5D and ERK5. These results suggest that the A-type current inactivation was inhibited by the ERK5 activation, regulating membrane excitability.

## 3. Discussion

**Figure 5:**
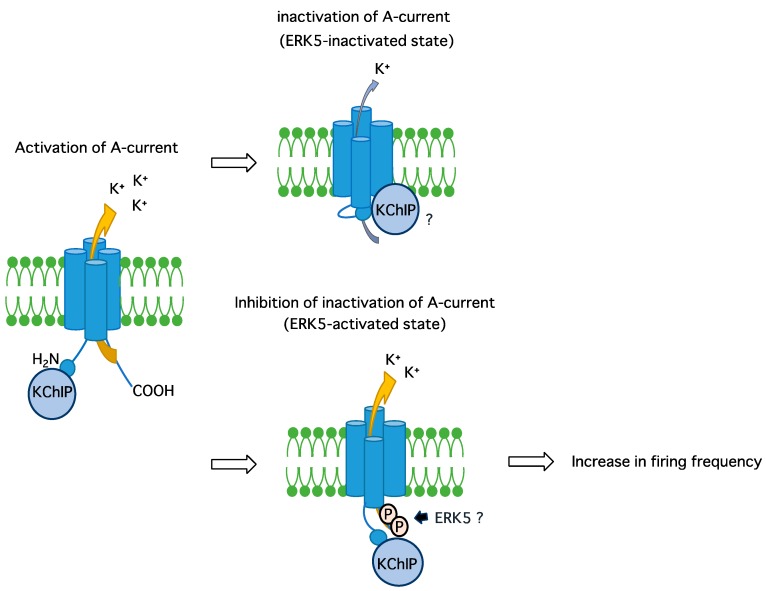
Putative mechanism of regulation of K_v_4.2 channels by ERK5. The ERK5 phosphorylates unidentified Ser/Thr residue(s) on K_v_4.2, resulting in the inhibition of the A-type current inactivation. This mechanism may contribute to rapid repolarization toward resting potential, which is necessary for causing the next firing.

In the present study, we found that ERK5 signaling promoted the mRNA expression of the K_v_4.2 primary subunits that underlie the transient A-type current in PC12 cells. However, its protein levels were not reflected by this mRNA up-regulation. Instead, the phosphorylation of endogenous K_v_4.2 proteins was promoted by ERK5 and the inactivation rate of the A-type current decreased in these cells. This putative mechanism is shown in Figure 5.

It has been demonstrated that the ERK5 knock-down by antisense oligonucleotides suppressed levels of transient receptor potential (TRP) V1 and A1 in dorsal root ganglion neurons [28]. In the study above, ERK5 regulated the TRPV1 and TRPA1 expression by an unknown mechanism. It has been shown that various transcription factors bind to the K_v_4.2 promoter and regulate the transcription. For example, GATA4 and 6, as well as FOG2 enhance K_v_4.2 transcription in PC12 cells, although there is a possibility that these transcription factors influence indirectly, as the GATA-binding consensus sequence is lacking in the minimum K_v_4.2 promoter [29]. Another study shows that the calcineurin/nuclear factor of the activated T cells (NFAT) pathway increases the K_v_4.2 mRNA and protein expression and promoter activity, without affecting the KChIP2 and K_v_4.3 levels in rat neonatal ventricular myocytes [30]. Furthermore, neuritin increases the A-type current density accompanied by the up-regulation of K_v_4.2 mRNA and protein via the Ca^2+^/calmodulin/calcineurin/NFATc4 and ERK/NFATc4 pathways in the central neurons, and affects neuronal excitability with increased dendritic spine formation [21]. Because there are NFAT binding sites in the K_v_4.2 promoter [21], ERK5 may phosphorylate NFAT to promote K_v_4.2 transcription, as ERK1/2 activation resulted in phosphorylation of Ser676 on NFATc4 [31]. However, the reason remains unknown as to why the K_v_4.2 protein levels were not reflected by its mRNA expression in this study. In contrast, ERK5 phosphorylation of K_v_4.2 was promoted without changes in the protein expression levels. This may reflect the results obtained by electrophysiological experiments that the peak current was not altered by the ERK5 activation. Furthermore, it is also reasonable that the change in the time constant of the A-type current was affected by the changes in K_v_4.2 phosphorylation status, but not the protein levels. 

It has been shown that ERK1/2 directly phosphorylates Thr602, Thr607, and Ser616 residues at the C-terminal cytoplasmic domain of K_v_4.2 [26]. These amino acids are entirely preserved among human and rat K_v_4.2. In this study, epidermal growth factor enhanced phosphorylation of K_v_4.2 at these three sites in COS7 cells overexpressing K_v_4.2. Although this phosphorylation was attenuated by U0126, which blocks ERK1/2 signaling, the remaining phosphorylated band was still observed. Because epidermal growth factor can activate both ERK1/2 and ERK5 [5,23], the remaining U0126-resistant K_v_4.2 phosphorylation component may result from ERK5 activity. This group further examined the effects of these three phosphorylated amino acids on the A-type current [24]. The mutation of these three amino acids to Asp caused the activation curve to shift toward more depolarized membrane potentials, whereas the mutation of these three amino acids to Ala showed no effect. Interestingly, the site-directed T607D mutant caused a rightward shift of the activation curve only in the presence of KChiP3, as observed in the case of the triple D mutant, but the S616D mutant caused a leftward shift, which is the totally opposite effect. It has been shown that ERK1/2 also phosphorylates K_v_4.2, reducing its conductance in neurons [18]. The minimum consensus sequence of ERK5 and ERK1/2 is similar, but ERK5 may preferentially phosphorylate the Ser616 residue, which results in rapid repolarization to increase the firing frequency, as described below. In contrast, the pituitary adenylate cyclase-activating polypeptide (PACAP) down-regulates the A-type current density without influencing the voltage-dependence of the Kv4.2 channel currents by ERK1/2 phosphorylation of K_v_4.2 in rat hippocampal neurons [19]. However, the characteristics of the site-directed mutants of the three amino acids above (T602A, T607A, and S616A) are different from the results found by the group mentioned above. The K_v_4.2 S616A mutant did not show any pituitary adenylate cyclase-activating polypeptide (PACAP) induced reduction in the channel current density, whereas the overexpression of T607A mutants partially blocked the inhibitory effect of PACAP. Additionally, the mutational analysis of K_v_4.2 indicates that Ser616 is the functionally relevant ERK1/2 phosphorylation site for the modulation of the K_v_4.2-mediated currents in neurons derived from spinal cord dorsal horns [20]. Therefore, the roles of the ERK phosphorylation site at the K_v_4.2 C-terminus are still controversial. Further study is necessary to identify the ERK5 phosphorylation site on K_v_4.2, and to examine the effect on the A-type current.

Adjusting the classical Hodgkin–Huxley models, Rush and Rinzel studied the effects of the A-current on the steady firing rate of neurons. They showed that the number of spikes per burst increases as the conductance of the A-current decreases and as inactivation decreases [32]. When ERK5 was activated, the A-current inactivation rate was reduced in our results (Figure 4). According to their model, we assume that ERK5 may contribute to more rapid repolarization toward the resting potential for responding to the next firing. Therefore, ERK5 may increase the firing frequency through the phosphorylation of K_v_4.2.

In conclusion, this study revealed, for the first time, that ERK5 signaling promotes phosphorylation of K_v_4.2 and inhibits the inactivation of the A-type current for the enhancement of membrane excitability in PC12 cells. ERK5 promotes neurite outgrowth and catecholamine biosynthesis. In addition to these roles, the regulation of membrane excitability may be essential for the differentiation process toward mature neurons. Future directions are examining the role of the ERK5-enhanced A-type current in neuronal morphological changes and functions in primary cultured neurons, using ERK5 conditional knockout mice. 

## 4. Materials and Methods

Materials: HRP-conjugated anti-glyceraldehyde-3-phosphate dehydrogenase (GAPDH) antibody was purchased from Cell Signaling Technology (Beverly, MA, USA). Anti-K_v_4.2 antibody was purchased from UC Davis/NIH NeuroMab (Davis CA, USA), and HRP-conjugated anti-mouse IgG secondary antibody was purchased from GE Healthcare (Buckinghamshire, UK). Enhanced chemiluminescence (ECL) assay kits were purchased from either GE Healthcare, PerkinElmer (Waltham, MA, USA) or Nacalai Tesque (Kyoto, Japan). Lipofectamine 2000 was purchased from Invitrogen (Grand Island, NY, USA). Mn^2+^-Phos-tag was purchased from Wako Pure Chemicals (Osaka, Japan). TriPure Isolation Reagent for the total RNA extraction, and the FastStart Essential DNA Green Master for real-time PCR were purchased from Roche (Indianapolis, IN, USA), and a Reverse Transcription kit was purchased from Toyobo (Osaka, Japan). A DNA plasmid encoding enhanced green fluorescent protein (EGFP) was purchased from Takara (Tokyo, Japan). The DNA plasmid encoding a tandem MRE-driven firefly luciferase was kindly given by Ron Prywes (Columbia University, NY, USA), and MEK5D (S311D/T315D) was kindly given by Eisuke Nishida (Kyoto University, Japan). DNA plasmid encoding oncogenic RasG12V mutant was used to activate ERK1/2. It was kindly given from Philip J.S. Stork (Vollum Institute, Oregon Health Sciences University, OR, USA). Because these DNA plasmids were kind gifts, as described above, there is restriction for the availability of these plasmids. ERK5KD (K83M) mutant was created from wildtype ERK5 as a template, as described previously [5]. 

Cell lines: The HEK293 cells and PC12 cells are provided by the Department of Cellular Signaling, Graduate School of Pharmaceutical Sciences, Tohoku University, Japan. Results using these cell lines have been published [9,23]. The HEK293 cells were grown in Dulbecco’s modified Eagle’s medium (DMEM), supplemented with 10% fetal bovine serum, penicillin (50 units/mL), and streptomycin (50 μg/mL), in a 5% CO_2_ incubator at 37 °C. The PC12 cells were grown in DMEM, supplemented with 10% FBS, 5% horse serum, penicillin (50 units/mL), and streptomycin (50 μg/mL) in a 5% CO_2_ incubator at 37 °C.

qRT-PCR: The total RNA from the PC12 cells was extracted using TriPure isolation reagent according to the manufacturer’s protocol. The RNA was then reverse transcribed using a RT-PCR kit, and real-time PCR was performed using a LightCycler Nano thermal cycler (Roche), as described previously [33]. The PCR primers used in the PC12 cell experiments were as follows: Ca_v_1.2 (5′-TGT TTC CAG ATG AGA CCC GC-3′ and 5′-GAG GCC CTT CGA CCT AGA GA-3′), Ca_v_2.1 (5′- CTG CTT TGA AGA GGG GAC AG-3′ and 5′-GGA AAA CAG TGA GCA CAG CA-3′), Ca_v_2.2 (5′-TCA TTG TGG TCT TCG CTC TG-3′ and 5′-CCT TTG CTG ACT CCT CCT TG-3′), K_v_4.2 (5′-TTG GCG ACT GCT GTT ATG AG-3′ and 5′-TGA CTG AGA CGG CAA TGA AG-3′), K_v_4.3 (5′-GGC TAC ACC CTG AAG AG CTG-3′ and 5′-GCC AAA TAT CTT CCC AGC AA-3′), KChIP3 (5′-GCC TTC GAT GCT GAT GGG AA-3′ and 5′-AGA GGT GCG TCC TTT CGC AG-3′), and GAPDH (5′-ACC ACA GTC CAT GCC ATC AC-3′ and 5′-TCC ACC ACC CTG TTG CTG TA-3′). PCR products were quantified and normalized to the GAPDH control before finally being presented as a fold change. 

Reporter gene assay: Reporter gene assays were performed similarly, as described previously [33]. MEK5D, ERK5, and MRE-luciferase reporter genes were co-transfected into HEK293 cells in 24-well plates using Lipofectamine 2000. Two days after transfection, the lysates were collected and the luciferase activity was measured using a luminometer (Lumat LB9507, Berthold Japan K.K., Tokyo, Japan). 

SDS-PAGE with or without Phos-tag and Western blotting: The proteins were separated by electrophoresis using 10–11% polyacrylamide gels. The proteins were then transferred from the gel onto a polyvinylidene difluoride membrane (GE Healthcare), according to standard protocols. The membranes were blocked for 0.5 h at room temperature in 5% skim milk in Tris-buffered saline containing 0.1% tween-20 (TBST), then incubated with the indicated primary antibodies overnight at 4 °C. The antibodies were dissolved in the blocking buffer, and used at the following dilutions: anti-K_v_4.2 (1:500 or 1:1000), and HRP-conjugated anti-GAPDH (1:1000). The membranes were washed several times with TBST before being incubated with HRP-conjugated anti-mouse IgG secondary antibodies (diluted 1:5000 in blocking buffer) at room temperature for 1–2 h. The membranes were then washed with TBST, developed using an ECL chemiluminescence assay kit, and visualized using a ChemiDoc XRS imaging system (BioRad, Hercules, CA, USA) or LAS1000 (Fuji Film, Tokyo, Japan). The relative intensities of the bands corresponding to K_v_4.2 and the internal control GAPDH were determined using Image-J densitometry software (National Institute of Health, Bethesda, MD, USA). 

For electrophoresis using Phos-tag, the proteins were separated with 5% polyacrylamide gels containing 30 μM Phos-tag and 60 μM MnCl_2_. After the gels were washed twice for 10 min with transfer buffer containing 10 mM EDTA to remove Mn^2+^, the proteins were then transferred from the gel onto a polyvinylidene difluoride membrane at 30 V for 16 h. The further procedure is performed similarly, as described above.

Electrophysiology by patch-clamping: The PC12 cells were co-transfected with EGFP, MEK5D, and ERK5. EGFP was used as a marker for the transfected cells. The whole-cell patch clamp method was used for recording the membrane currents (patch-clamp amplifier Axopatch 200B, Molecular Devices, Chicago, IL, USA), as described previously [34]. Borosilicate glass electrodes had tip resistances between 2.5 and 4.5 MΩ when filled with internal solution composed of (mM) KOH 120, aspartic acid 80, Mg-ATP 5, KCl 20, HEPES 5, EGTA 5, and GTP-Na_2_ 0.1 (pH 7.2 with aspartic acid). The composition of the external solution (mM) was: NaCl 136.9, KCl 5.4, CaCl_2_ 1.8, MgCl_2_ 0.5, NaH_2_PO_4_ 0.33, HEPES 5.0, and glucose 5.5 (pH 7.4 with NaOH). To evoke membrane currents, the cells were held at a potential of −80 mM and depolarized for 500 ms to various potentials, ranging from −30 to +50 mV in 20 mV increments at 37 ± 0.5 °C. The pulse protocol and data acquisition and storage were accomplished with Clampex 9.2 (Molecular Devices). The sampling frequency was 10 kHz and low-pass filtering was performed at 5 kHz. The cell membrane capacitance (C_m_) was determined by integrating the area under the capacitive transient elicited, by applying a 50 ms hyperpolarizing voltage-step from a potential of −40 to −45 mV. All membrane currents (I_m_) were normalized by Cm, then analyzed using IGOR software (Wavemetrics, Portland, OR, USA). The time-course of inactivation at 50 mV was fitted with a first order biexponential function, as follows:

$$I_{m}\left(t\right)=y_{0}+y_{1}\left\{1-\exp\left(-\frac{t}{\tau_{fast}}\right)\right\}+y_{2}\left\{1-\exp\left(-\frac{t}{\tau_{slow}}\right)\right\}$$

where *τ_fast_* and *τ_slow_* are fast and slow time constants, respectively.

Statistics: Data are expressed as means ± S.E.M., and the statistical significance of the differences between groups was analyzed using the unpaired Student’s *t*-test or one-way ANOVA, with post hoc test using Tukey’s test for multiple comparisons.

## Abbreviations

MAPK	Mitogen-activated protein kinase
ERK	Extracellular signal-regulated kinase
MEK	MAPK/ERK kinase
MEF	Myocyte-enhancer factor
MRE HEK293 cells	MEF2 response elementhuman embryonic kidney 293 cells
SDS-PAGE	Sodium dodecyl sulfate-polyacrylamide gel electrophoresis
TRP	Transient receptor potential
NFAT	Nuclear factor of activated T cells
KChIPCA	K^+^ channel-interacting proteincornu ammonis
PACAP	Pituitary adenylate cyclase-activating polypeptide
GAPDH	Glyceraldehyde-3-phosphate dehydrogenase
ECL	Enhanced chemiluminescence
EGFP	Enhanced green fluorescent protein
DMEM	Dulbecco’s modified Eagle’s medium
TBST	Tris-buffered saline containing 0.1% tween-20
